# Early Diagnosis and Early Intervention in Cerebral Palsy

**DOI:** 10.3389/fneur.2014.00185

**Published:** 2014-09-24

**Authors:** Mijna Hadders-Algra

**Affiliations:** ^1^Department of Pediatrics – Developmental Neurology, University Medical Center Groningen, University of Groningen, Groningen, Netherlands

**Keywords:** early diagnosis, early intervention, cerebral palsy, neuroplasticity, general movements assessment

## Abstract

This paper reviews the opportunities and challenges for early diagnosis and early intervention in cerebral palsy (CP). CP describes a group of disorders of the development of movement and posture, causing activity limitation that is attributed to disturbances that occurred in the fetal or infant brain. Therefore, the paper starts with a summary of relevant information from developmental neuroscience. Most lesions underlying CP occur in the second half of gestation, when developmental activity in the brain reaches its summit. Variations in timing of the damage not only result in different lesions but also in different neuroplastic reactions and different associated neuropathologies. This turns CP into a heterogeneous entity. This may mean that the best early diagnostics and the best intervention methods may differ for various subgroups of children with CP. Next, the paper addresses possibilities for early diagnosis. It discusses the predictive value of neuromotor and neurological exams, neuroimaging techniques, and neurophysiological assessments. Prediction is best when complementary techniques are used in longitudinal series. Possibilities for early prediction of CP differ for infants admitted to neonatal intensive care and other infants. In the former group, best prediction is achieved with the combination of neuroimaging and the assessment of general movements, in the latter group, best prediction is based on carefully documented milestones and neurological assessment. The last part reviews early intervention in infants developing CP. Most knowledge on early intervention is based on studies in high-risk infants without CP. In these infants, early intervention programs promote cognitive development until preschool age; motor development profits less. The few studies on early intervention in infants developing CP suggest that programs that stimulate all aspects of infant development by means of family coaching are most promising. More research is urgently needed.

## Introduction

Cerebral palsy (CP) is a common neuropediatric disorder with a prevalence of about 2‰ in high-income countries (1) and presumably higher prevalences in lower income countries (2). CP describes a group of disorders of movement and posture. Or, according to the internationally recognized definition of Rosenbaum et al. (3), “cerebral palsy describes a group of developmental disorders of movement and posture, causing activity restrictions or disability that are attributed to disturbances occurring in the fetal or infant brain. The motor impairment may be accompanied by a seizure disorder and by impairment of sensation, cognition, communication, and/or behavior.” The definition includes the notion that CP originates during early development, i.e., prenatally or relatively early postnatally. Even though the upper age limit of the postnatal time window is debated (4), CP mostly originates from an event occurring before the age of 6 months corrected age (CA).

The definition of CP highlights the diversity of neural impairments involved in CP, while simultaneously underlining the implications of the impairments for activities and participation. Nowadays, the major goal of rehabilitation services is to optimize home and community participation (5), implying that clinical management comprises all aspects of the framework of the international classification of functioning, disability and health, child and youth version [ICF-CY (6)]. As a result, clinicians working in the field of neuropediatrics and pediatric rehabilitation need to understand topics varying from neurodevelopmental mechanisms to family function. The aim of the present paper is to briefly review and critically discuss (a) prenatal and early postnatal brain development, the effect of an early lesion of the brain, and the consequences of neurodevelopmental principles for early diagnosis and early intervention in CP, (b) tools for early diagnosis, and (c) early intervention.

## Prenatal and Early Postnatal Brain Development

### Intricate processes of brain development

The development of the human brain is an intricate and long-lasting process. This is particularly true for the development of the neocortical circuitries; it takes about four decades time before they have established their “adult” configuration (7). Here, I will primarily discuss the developmental processes occurring in the prenatal and early postnatal period and I will focus on the neocortex and cerebellum, the structures where the vast majority of human neurons can be found (8). First, neocortical development is described. This description also serves to illustrate the complex and only partially understood developmental processes in the brain. Next, cerebellar development is discussed.

#### Development of the neocortex

**Figure 1:**
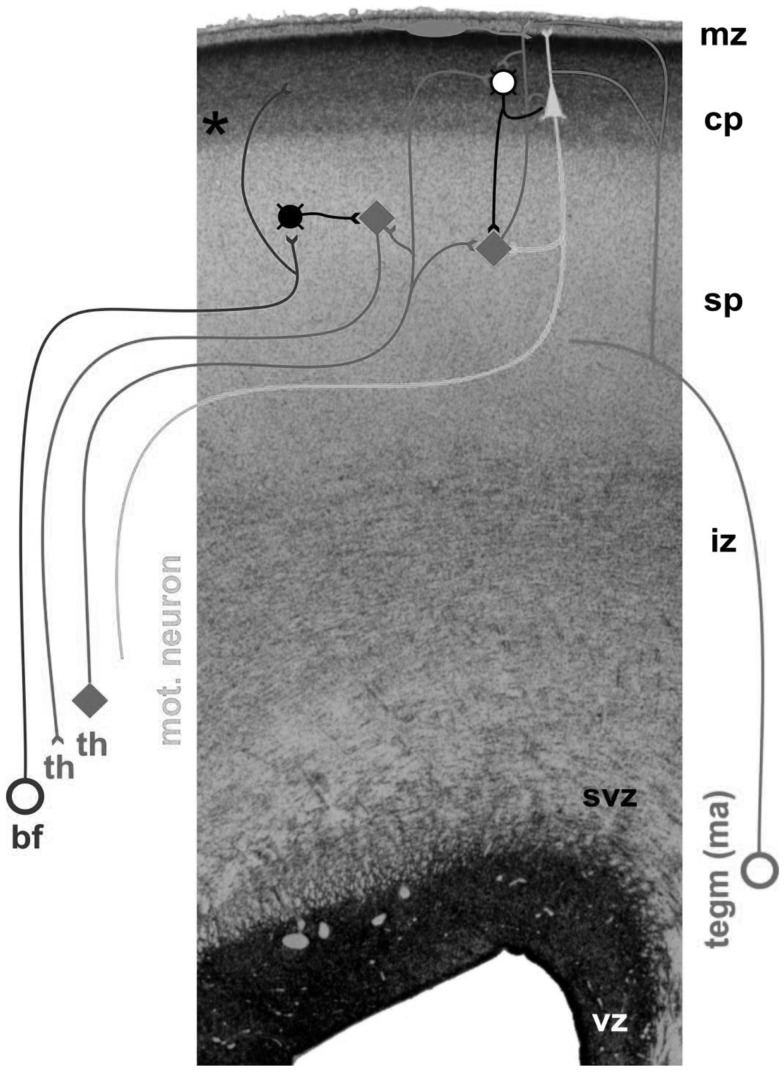
**Cross-section through the cortex of a fetus of 24 PMA**. The following layers can be distinguished, from the inside (bottom) to the outer surface (top): vz, the ventricular zone, which produces neurons; svz, the subventricular zone, which possibly is phylogenetically younger than the ventricular zone, and which produces neurons and glial cells (14); iz, the intermediate zone, i.e., the future white matter; sp, the subplate, which at this stage is very thick and harbors the transient fetal circuitry; cp, the cortical plate; mz, the marginal zone. Ingrowing afferents come from the basal forebrain (bf), thalamus (th), and monoaminergic brain stem nuclei (tegm ma). Figure by curtsy of Dr. Ivica Kostovic, University of Zagreb.

Neocortical development starts during the early phases of gestation with the proliferation of neurons. The majority of telencephalic neurons are produced in the first half of gestation in the germinal layers near the ventricles (9, 10). Young neuroblasts move from their place of origin to their final place of destination in the more superficially located cortical plate (9, 11). Neural migration is guided by the shafts of transient radial glial cells (10). However, initially developmental focus does not center on the cortical plate, but on a temporary structure, i.e., the subplate [Ref. (8), Figure 1]. The subplate is situated between the cortical plate and the intermediate zone, i.e., the future white matter (12). It contains a variety of neurons, most of which are glutamatergic (13). The subplate is thickest around 29 weeks postmenstrual age (PMA), when it is about four times thicker than the cortical plate (8). Thereafter, the subplate gradually disappears during the perinatal and early postnatal period, although it remains present below the associative cortices up to 6 months post-term (8).

Neurons start to differentiate during migration. Neuronal differentiation includes the formation of dendrites and axons, the production of neurotransmitters and synapses, and the elaboration of the intracellular signaling machinery and complex neural membranes (15, 16). The subplate, which became increasingly important during phylogeny (17), plays an important role in the processes of differentiation and cortical organization (8, 18). It is the major site of neocortical synaptogenesis. It also serves as a waiting and guidance compartment for growing cortical afferents, in particular, thalamocortical and corticocortical fibers. The cortical afferents “wait” for several months in the subplate before relocating from 28 weeks PMA onward into their final target, the cortical plate (8, 19). Evidence suggests that the ingrowing thalamocortical fibers meet the corticofugal projections of early-born preplate neurons. In other words, early corticofugal projections form “hand-shaking” scaffolds for the ingrowing thalamocortical fibers (20). During their fetal presence, the diverse and transient circuitries of the subplate are a prominent site of synaptic interaction; the subplate neurons produce spontaneous activity, and process the sensory information of the thalomocortical fibers (8).

The processes of neural differentiation and cortical organization are particularly active in the few months prior to birth and the first postnatal months. Developmental processes in the subplate, i.e., in the cortical–subcortical interface, continue to play a prominent role in cortical organization. During this period, the human cortex is characterized by the co-existence of two separate but interconnected cortical circuitries; the transient fetal circuitries centered in the subplate and the immature, but progressively developing permanent circuitry centered at the cortical plate (8). The duration of the “double circuitry” phase differs for the various regions in the cortex. For instance, the final phase of permanent cortical circuitry is reached around 3 months postnatally in the primary motor, sensory, and visual cortices, but first around the age of 1 year in the associative prefrontal cortex (18).

Besides neural cells, glial cells are generated. The peak of glial cell production occurs in the second half of gestation. Glia cell production includes the generation of oligodendrocytes, the cells involved in axonal myelination. Oligodendrocyte development reaches its peak between 28 and 40 weeks PMA (13). Myelination takes place especially between the second trimester of gestation and the end of the first postnatal year. It occurs earlier in sensory pathways than in motor ones, and earlier in projection fibers than in associative fibers (21). Beyond infancy, myelination continues until the age of about 40 years when the last intracortical, in particular, the long-fronto-temporal connections such as the cingulum, complete myelination (22).

Brain development does not only consist of the creation of components but also of an elimination of elements. About half of the created neurons die off by means of apoptosis. Apoptosis is brought about by interaction between endogenous programed processes and chemical and electrical signals induced by experience (23). In the neocortex, apoptosis occurs, in particular, between 28 weeks PMA and term age (24). Not only neurons are removed but also axons and synapses are eliminated. A well-known example is the pruning and tuning of the corticospinal tract: during the last trimester of gestation and continuing in the first two postnatal years the initially bilateral corticospinal projections in the spinal cord are reorganized into a mainly contralateral fiber system (25). This reorganization is activity driven and use dependent, as is illustrated by the effect of an early unilateral lesion of the brain. The latter results in asymmetrical activation of the spinal cord, inducing a preferential strengthening of the activity from the ipsilateral projections from the contralesional hemisphere in comparison to the contralateral projections from the ipsi-lesional hemisphere (25, 26).

The elimination of synapses in the brain starts already during early development, but in the neocortex this process becomes especially prominent between the onset of puberty and early adulthood. As a result, developmental remodeling of cortical neuronal circuitries continues well into the third decade of life (27).

#### Development of the cerebellum

Both the classical studies of John Dobbing (28, 29) and modern imaging studies (30) revealed that the cerebellum develops at high speed between 24 and 40 weeks PMA. Cerebellar volume increases with a factor 3 and cerebellar surface – during the formation of the characteristic cerebellar “folia” – with a factor 30 (31). In 2009, Joseph Volpe excellently reviewed the developmental processes in the cerebellum (31). Below, I summarize his review.

**Figure 2:**
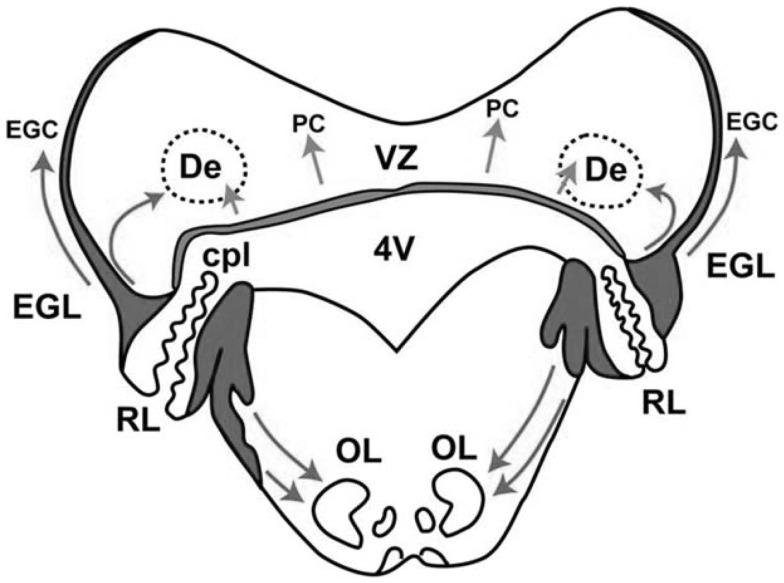
**Schematic representation of the two proliferative zones in the cerebellum around 14 weeks PMA, the dorsomedial ventricular zone (VZ), and the dorsolateral rhombic lip (RL)**. The VZ gives rise to the interneurons of the deep cerebellar nuclei, such as the dentate (De) and to Purkinje cells (PC). Migration occurs radially. The RL has two portions divided by the choroid plexus (cpl) of the 4th ventricle (4V). The upper portion gives rise to the granule precursor cells (EGC) of the external granular layer (EGL) – the cells initially migrate tangentially over the surface of the cerebellum. The tangential migration is later followed by an inward migration to the internal granular layer. The lower portion of the RL gives rise to neurons in the pons, including those of the inferior olive (OL). The arrows indicate the directions of migration. With permission from Dr. Joseph Volpe (31).

In the cerebellum, two proliferative zones can be distinguished: (a) the ventricular zone, which gives rise – by radial migration – to the deep cerebellar nuclei and the Purkinje cells, and (b) the rhombic lip, which gives rise – by tangential migration – to the external granular layer (Figure 2). The external granular layer is a transient structure that reaches its peak thickness between 20 and 30 weeks PMA. At that time, the cells of this layer (the granule cells) start to migrate inward – guided by Bergmann glial fibers – through the molecular layer with Purkinje cells, to their destination in the internal granular layer. During the inward migration, the granule cells form horizontal parallel fibers that contact the Purkinje cells. When the granule cells have arrived in the internal granular layer they soon receive input from the mossy fibers from the pons. Between 30 and 40 weeks PMA, the external granular layer is heavily involved in cell proliferation. It results in the previously mentioned fabulous expansion of the cerebellar surface. Meanwhile, the inward migration of the granule cells to the internal granular layer continues. In the first postnatal year, the external granular layer decreases in size and activity. Simultaneously, the internal granular and molecular layer increase in size. The latter is especially due to the elaboration of granule cell axons (parallel fibers) and Purkinje cell dendrites.

### Effect of an early lesion of the brain

Over the years, animal data have demonstrated that the effect of a lesion of the developing brain depends on the point in time at which the lesion occurred. Originally, it was thought that “the younger the age at insult, the better the outcome” [the so-called Kennard-principle (32)]. But gradually it became clear that this is not always true (33). Many factors determine the consequences of a lesion of the developing brain: the age at insult, the site, and the size of the lesion, its unilateral or bilateral nature, animal species, sex, exposure to chemical substances prior to and after the insult, and environmentally induced experience. Rodent studies indicated that, in particular, two types of environmental experience are associated with improved outcome: being raised in a complex environment and tactile stimulation at early age (33, 34). Animals with an early lesion of the brain who are raised in a complex environment, including attractive toys and peers, have a significantly better motor and cognitive outcome than lesioned animals brought up in a standard, “boring” laboratory environment. The improved functional outcome is associated with increases in brain weight, cortical thickness, and dendritic length. It has been suggested that part of the effect of the complex environment is mediated by increased maternal care in the form of licking and grooming, i.e., early tactile stimulation. Indeed, other studies revealed that tactile stimulation of pups, who acquired an early lesion of the brain, is associated with improved motor and cognitive outcome and increased dendritic spine density, changes that presumably are mediated by increased levels of neurotrophic factors (33). The complex picture emerging from the animal studies is that, each age, each neural system, each species, and each sex has specific vulnerabilities and resources of resilience to cope with the effects of an early lesion. Nevertheless, within the complexity three general principles may be distinguished: (a) bilateral lesions are associated with a lower potential for functional plasticity and with worse outcome than unilateral lesions; (b) large (unilateral) lesions are associated with less recovery and worse functional outcome than small (unilateral) lesions; (c) cognitive functions show a better recovery than motor functions (33).

Retrospective magnetic resonance imaging (MRI) studies in children with CP demonstrated that the most common brain lesion in these children is damage of the periventricular matter. A recent review of population-based studies carried out in western industrialized countries, revealed that a lesion of the periventricular white matter is present in 19–45% of children with CP (35). Other relatively frequent lesions are gray matter injury, including lesions of the cortical gray matter, the basal ganglia, and the thalamus (21%), malformations (11%), and focal cortical infarcts (10%) (35). Note that in about 15% of children with CP structural MRI scans do not show abnormalities (35, 36). The varied MRI findings illustrate the neurodevelopmental heterogeneity of CP. However, the findings do not inform us about the neural mechanisms operating when the brain acquires a specific lesion at a certain early age. These mechanisms may involve plastic, restorative adaptations, but they also may result in deleterious changes.

Below, I summarize the neurodevelopmental sequalae of two major categories of brain lesions: damage of the periventricular white matter and unilateral lesions of the brain.

#### Damage of the periventricular white matter

Lesions of the periventricular white matter mostly originate between the ages of 24 and 34 weeks PMA. Prospective imaging studies on the developmental sequelae of damage of the periventricular white matter indicated that focal necrotic lesions [cystic periventricular leukomalacia (PVL)] are associated with a high risk for CP [>80% (37, 38)]. The risk for CP is higher in posterior than in anterior lesions (39). In addition, the severity of CP following PVL depends on the severity of the cystic lesion: focal cysts generally give rise to bilateral CP with a diplegic distribution and more extensive cysts result in bilateral CP with a quadriplegic distribution (39). In fact, the cystic lesions are the tip of the iceberg of the pathology in the periventricular white matter, as the cystic lesions are surrounded by diffuse astrogliosis and microgliosis in the white matter (13, 40). Actually, in modern neonatal intensive care units (NICUs) cystic PVL accounts for only a minority of the lesions of the periventricular white matter. Much more common is the “non-cystic PVL,” which consists of diffusely distributed, small lesions of the periventricular white matter (13). The specific characteristics of non-cystic PVL in neonatal imaging are debated [ultrasound, periventricular echodensities (38); MRI, punctate lesions and diffuse excessive high-signal intensity (39)]. Notwithstanding the variation in criteria for non-cystic PVL, the data indicate that non-cystic PVL is also associated with CP, be it with lower risk rates than cystic PVL (38).

Brain pathology in children with PVL is, however, not restricted to the periventricular white matter of both hemispheres. PVL is accompanied by a half to three-quarter reduction in the number of the most prevalent type of neurons in the subplate, i.e., the polymorphic non-pyramidal and inverted pyramidal neurons. This reduction does not only occur at the site of the lesion but also in remote areas (40). PVL also is associated with a bilateral decrease in cerebellar volume (31), an altered arborization of the noradrenergic fibers in the cerebellar circuitries (41), and – in a substantial proportion of children – with neuronal loss in the thalamus and basal ganglia (13). The associated pathologies may be the result of the hypoxic-ischemic and inflammatory events that caused the PVL, but they may also be the result of diaschisis, i.e., the loss of function in a neurally connected region (31). More recently, it has been suggested that the injury processes that cause PVL, including persistent inflammation and epigenetic changes, may persist for many months or even years (42). The latter implies that the well-known “growing into a deficit” principle manifested during the development of a child with CP (33, 43) may not only be caused by the age-related development of increasingly complex cerebral functions and therewith the expression of impairments in these functions but may also be the result of progressive damage. The widespread encephalopathy of PVL explains why PVL often results in bilateral CP that is often associated with cognitive impairments (44). Unfortunately, the widespread bilateral brain damage and its motor and cognitive sequelae limit the possibilities of early intervention to induce beneficial plastic changes in the brain (33).

#### Unilateral lesions of the brain

Basically, two types of unilateral lesions of the brain can be distinguished: (a) unilateral periventricular hemorrhagic infarction, occurring in preterm infants of 24–34 weeks PMA, and involving the periventricular white matter [further referred to as “preterm” lesions (45–47)], and (b) focal cortical–subcortical infarction, occurring around term age, and usually affecting the area of the medial cerebral artery (further referred to as “term” lesions). The term lesion in general does not involve the periventricular white matter (47, 48). These two lesions may be regarded as the two ends of the developmental spectrum of perinatally acquired unilateral lesions of the brain. In clinical life also mixed patterns exist. In addition, the lesions may also occur bilaterally – the ratio between unilateral and bilateral forms being 3:1 (46, 48). Below, I will discuss the sequelae of the unilateral lesions in children who develop CP. It should be realized, however, that about 25–50% of the infants with a perinatally acquired unilateral lesion of the brain does not develop CP (48–51). The children with a unilateral brain lesion, who do develop CP, mostly present with unilateral CP, but some infants develop bilateral CP (52).

Staudt and co-workers demonstrated in children with unilateral CP that the plastic events in response to a unilateral lesion do not only vary with the timing of the lesion but also with the neural system involved (47, 53). In the motor system, the reorganization may involve persistence of the typically transiently present ipsilateral corticospinal projections. The chance that ipsilateral projections persist increases with decreasing gestational age. As a result, the function of the paretic hand in children with unilateral CP resulting from a preterm lesion often involves ipsilateral corticospinal activity, and the function of the paretic hand in children with unilateral CP resulting from a term lesion generally involves contralateral corticospinal activity (25, 47). However, many mixed patterns exist. In general, the presence of more ipsilateral projections is associated with worse bimanual function (54). In part, the worse bimanual function may be due to hindering “mirror movements,” as motor control of both hands is mediated by the same corticospinal system (25, 47).

For the sensory system, the effect of a preterm unilateral lesion differs from that occurring in the motor system. In the sensory system, reorganization does not involve structures on the unaffected side of the brain. It is mediated by structures on the lesioned side (47, 53, 55). This reorganization is related to the way in which the sensory system develops at early preterm age. At that time, the ascending thalamocortical somatosensory projections have not yet reached the cortex, allowing the ingrowing axons to take a detour, and bypass the lesion in order to reach the cortex. This axonal plasticity is associated with good or only minimally reduced somatosensory function. In term unilateral lesions, such reorganization is no longer available. Consequently, lesions often result in severe somatosensory deficits (47).

**Figure 3:**
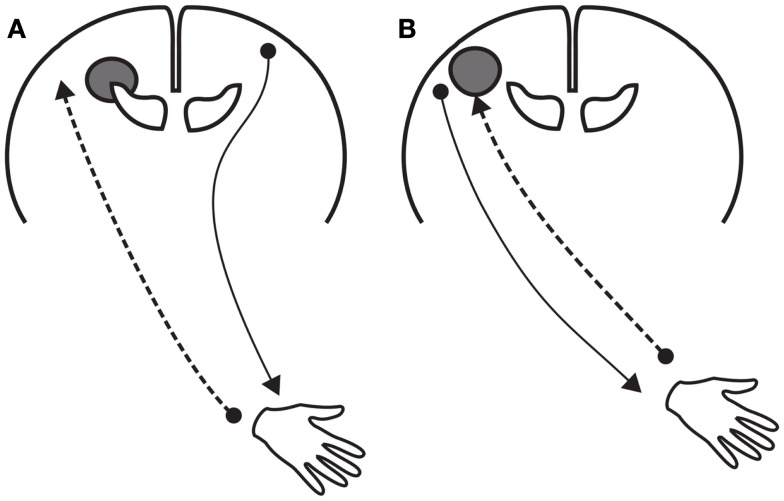
**Schematic representations of the reorganization of motor and sensory function after a unilateral lesion of the brain at early age**. **(A)** Reorganization after a “preterm” unilateral lesion. The lesion involves the periventricular white matter. The reorganization includes (a) the persistence of ipsilateral corticospinal projections to the paretic hand originating in the contralesional hemisphere and (b) axonal plasticity of the thalamocortical afferents bypassing the lesion in the ipsi-lesional hemisphere. **(B)** Reorganization after a “term” unilateral lesion, which usually does not include the periventricular white matter. Motor and sensory functions of the paretic hand are organized in the lesioned hemisphere.

The differential reorganization between motor and sensory functions imply that sensorimotor control in children with unilateral CP resulting from a preterm lesion differs from that resulting from a term lesion (Figure 3). In children with a typical preterm lesion control is dissociated; somatosensory information of the paretic hand is processed by the lesioned, contralateral hemisphere, but motor commands for the paretic hand are generated in the non-lesioned, ipsilateral hemisphere (53, 56). This neurophysiological make-up is usually associated with a relatively intact processing of sensory information in combination with moderate motor control. In children with a term lesion, somatosensory processing and motor control of the paretic hand both occur in the lesioned, contralateral hemisphere. Manual ability in these children varies considerably, and presumably is largely affected by the substantial somatosensory deficits (47, 57).

The language system is characterized by yet another form of plasticity (47, 56). In the majority of human adults, language develops predominantly in the left hemisphere. In infants, who perinatally acquire a lesion of the left hemisphere, the language system may move entirely to the homotopic area in the right hemisphere, with no or little loss of function (47). This happens more often in infants with a term lesion than in those with a preterm lesion (58).

### Implications for early diagnostics and early intervention

The population-based MRI review of Reid and colleagues (35) indicated that in western industrialized countries 50–75% of children with CP acquire their lesion between 24 weeks PMA and term age. This is the period during which brain development is characterized by a high rate of widespread and complex developmental processes. The rapid changes over time do not only induce age-specific vulnerabilities of the brain, e.g., lesions in the periventricular white matter at early preterm age and lesions in the cortical–subcortical areas around term age, but also induce age-related plasticity. As a consequence, early neurological dysfunction after preterm lesions may be expressed in a different way than that after term lesions. For instance, during the first months post-term infants with a preterm lesion of the brain developing CP may present with more or less typical muscle tone and reflexes in combination with an abnormal quality of general movements, whereas infants with a term lesion of the brain developing CP usually present with dysfunction in all aspects of neurological function, i.e., in muscle tone, reflexes, and the quality of general movements (59–61).

Relatively little data are available on the prevalence and the etiology of CP in low- and middle-income countries (LMIC). The limited LMIC-data available suggest that CP in these countries is less often caused by complications associated with preterm birth than in western industrialized countries, and more often by asphyxia and hyperbilirubinemia at term, and by postnatal infections, such as meningitis (62–64). This means that the presentation of CP in LMIC differs from that in western industrialized countries. The differences involve different etiological mechanisms, a different timing of the lesion and different plastic changes of the brain. This may mean that knowledge on early diagnostics and early intervention coming from western industrialized countries may not immediately be generalized to LMIC.

Early diagnostics and early intervention after a perinatal lesion of the brain occur especially in the preterm period and during the first year post-term. During this period, the double neocortical circuitry, in which the subplate and cortical plate circuitries co-exist, is gradually substituted by the single circuitry of the developing networks in the cortical plate. Also, the cerebellum shows dramatic developmental changes during this period. For diagnostics, these large neurobiological changes have major consequences. First, the fact that a child has an age-specific nervous system invokes the need of age-specific assessments, that is, evaluation techniques, which are adapted to the age-specific characteristics of the nervous system. Examples are age-specific neurological, motor, and cognitive assessments. Apart from the need of age-specific instruments, assessments are in need of age-specific norms. This is not only true for functional assessments, such as neurological exams and cognitive tests, but also for imaging techniques and physiological assessments. Second, the age-dependent characteristics of the nervous system affect the way in which neural dysfunction is expressed. Neurological dysfunction in adults is expressed by means of specific and localized signs, e.g., by means of the specific syndrome of a spastic hemiplegia in case of stroke. In contrast, neurological dysfunction in young infants is expressed by means of generalized and unspecific dysfunction. For instance, a preterm infant with a left-sided infarction may respond with generalized hypotonia, generalized hypertonia, hypokinesia, a hyperexcitability syndrome, or with abnormal general movements (43, 65). In infants developing CP, the early unspecific neurological dysfunction gradually develops into the specific syndrome of CP. This development may take 1–5 years, but in most children the diagnosis can be established by the age of 18–24 months (4). Third, the marked developmental changes of the brain have important implications for the prediction of developmental disorders at early age. The plastic changes may induce a disappearance of dysfunctions present at early age – infants grow out of their deficit. The reverse is also possible: children may be virtually free from signs of dysfunction at early age, but grow into a functional deficit with increasing age due to the age-related increase in complexity of neural functions (66).

The amazing developmental changes of the brain between preterm age and the age of 1 year post-term offer opportunities for early intervention. Animal studies indicated that intervention has the largest effect when applied during the period when dendrites and synapses are produced at a high rate. The period during which the double cortical circuitry configuration wanes offers therefore large opportunities for early intervention. On the other hand, it should be realized that the early lesions themselves often are associated with pathological processes elsewhere in the nervous system, either as a remote consequence of the primary lesion or as a concomitant effect of the harmful events that caused the primary lesion. In addition, the prenatal and perinatal complications, resulting in the lesion of the brain may also imply the presence of a prolonged period of stress and pain for the young infant. Stress during early life is known to have lifelong consequences, as it induces permanent changes in the brain, especially in the mono-aminergic systems, such as the dopaminergic system (67, 68). Alterations in the dopaminergic system are associated with impaired motor learning (69). Indeed, it has been demonstrated that preterm infants with and without lesions on the ultrasound scan of the brain have deficits in motor learning. They have more difficulties than typically developing term infants to build internal reference frames of body configuration on the basis of daily experience. As a result, they rather rely on simple feedback motor control strategies than on feedforward motor control (70). Unfortunately, the presence of widespread neural impairment in the brain reduces the potential for plastic changes of the young nervous system, as animal experiments indicated that the chance of beneficial effects of intervention decreases with the extent of brain pathology (33, 34).

## Early Diagnostics in CP

The preceding paragraph stressed that the developmental changes of the brain hamper prediction of CP at early age. This does, however, not imply that we are totally at loss with prediction. Prediction improves when we use multiple tools, such as neuroimaging, neurological and neuromotor exams, and neurophysiological assessments (71–73). Prediction also improves substantially when longitudinal series of assessments are used – developmental trajectories predict developmental outcome best (74–76). Knowledge on the predictive value of single assessments with specific diagnostic instruments is generally based on selective groups of high-risk infants in western industrialized countries (77). This means that the findings may neither be generalized to populations of high-risk infants in LMIC settings nor to general populations across the world, which mostly consist of low-risk infants. In this respect, it is also important to note that the Surveillance of CP in Europe study revealed that 70% of children with CP had been admitted to a special care infant unit after birth (78). Recall too that the retrospective imaging study of Reid et al. (35) indicated that 50–75% of brain lesions occur between 24 weeks PMA and term age. Both types of data suggest that 25–50% of children with CP will not show signs suspect for CP during the newborn period, and thus, will not receive neonatal monitoring to predict developmental outcome. In the following sections, I will address the assessment methods that are most frequently used in the early prediction of CP: (a) neurological and neuromotor assessments, (b) neuroimaging, and (c) neurophysiological tests.

### Neurological and neuromotor assessments

Neurological assessments are frequently used to monitor development of high-risk infants. The best known methods are the Dubowitz assessment for neonates (79) and its adaptation for older infants, the Hammersmith infant neurological examination [HINE (80)], the Prechtl assessment for newborns (65) and its adaptation for older infants, the Touwen infant neurological examination [TINE (75, 81)], and the assessment according to Amiel-Tison (82). Predictive validity of these assessments is generally good (82), with an estimated sensitivity and specificity for CP of 88–92%, respectively (77).

The best known neuromotor assessment is the general movement assessment (GMA). General movements are the most frequently used movements from early fetal age until 3–4 months post-term (83). The quality of these movements provides information about the integrity of the brain, possibly especially about the connectivity in the periventricular white matter (84). Typical general movements are characterized particularly by variation and complexity; in abnormal general movements these characteristics are reduced or absent (83, 84). Prediction of CP with the GMA is excellent when based on longitudinal series of assessments (84). When a single assessment is used, prediction is best when GMA is carried around 3 months post-term [median sensitivity 98% (range 50–100%), specificity 94% (range 35–100%) (77, 85, 86)]. Other motor assessments used to predict CP in infancy are the motor assessment of infancy [MAI (87)], the test of infant motor performance [TIMP (88)], the Alberta infant motor scale [AIMS (89)], the infant motor profile [IMP (90)], and – less frequently – the psychomotor index of the Bayley scales of development (91). However, calculation of predictive values for CP is precluded due to the limited data on prediction available, or due to the fact that studies evaluating the predictive properties used more global “abnormality” outcomes (77, 85, 92).

The neurological and neuromotor assessments are relatively cheap instruments, and therefore, may be applied in many settings across the world. Another instrument that may be used in low-risk populations and in settings with limited resources is the developmental assessment of young children [DAYC (76)]. The DAYC has a complementary approach: parental information on motor milestones serves as the starting point for a quick testing of the limits of the infant’s skills. The retrospective study of Maitre et al. (76) demonstrated that a decrease in DAYC-scores between 6 and 12 month of age was highly predictive of CP.

### Neuroimaging

Neurological condition in infants admitted to a NICU is virtually always evaluated by imaging of the brain, in particular, by cranial ultrasound (cUS) and MRI. Neonatal cUS is especially applied in preterm infants. It readily visualizes large lesions of the periventricular white matter, such as periventricular hemorrhagic infarction and cystic PVL. For proper prediction of outcome, it is recommended to make series of cUS, i.e., sequential cUS during the first 4–6 weeks after birth and an additional one at 36–40 weeks PMA, as it takes 2–5 weeks for cysts to develop (38). Meta-analysis of six studies including over 2400 preterm infants on the power of neonatal cUS to predict CP indicated an estimated sensitivity of 74% and an estimated specificity of 92% (77). cUS may also assist prediction of the type and severity of CP (93). Unilateral infarctions are associated with unilateral spastic CP (but recall that this is not a one-to-one relationship!). Deep gray matter lesions are associated with dyskinetic CP and severe impairment (93). In case of PVL, cUS also serves the prediction of the ability to walk independently at the age of 2 years: children with grades I and II PVL usually are able to walk at age 2, whereas <10% of children with grade III (extensive PVL) are able to do so (38).

Magnetic resonance imaging is the preferred imaging technique in (near) term infants with hypoxic-ischemic neonatal encephalopathy. It is also increasingly used in preterm infants. In the latter group, the best age for MRI is term equivalent age (TEA), i.e., 40–42 weeks PMA. Scans at earlier ages run the risk of missing relevant damage of the posterior limb of the internal capsule (38). Limited data on the precise prediction of CP with neonatal MRI are available. However, existing data suggest that high sensitivity and high specificity (38, 77). Currently, also more sophisticated MRI techniques are applied, such as diffusion-weighted imaging [to visualize hypoxic-ischemic brain lesions (94, 95)], diffusion tensor imaging [DTI, to visualize the microstructure of the white matter (96)], and magnetic resonance spectroscopy imaging [for detailed information on local brain metabolism (95)]. These instruments are promising, in particular, DTI of the posterior limb of the internal capsule (96), but details on prediction of CP are very limited.

### Neurophysiological tests

Neurophysiological tests are especially used in term infants with neonatal encephalopathy. The recent systematic review of Laerhoven et al. (95), which addressed the predictive value of various tests in infants who mostly suffered from moderate to severe neonatal encephalopathy, indicated that the amplitude-integrated electroencephalogram (aEEG) and traditional electroencephalogram (EEG) predicted outcome well. Abnormal outcome was, however, defined as either death or having a moderate to severe disability. More recently, the aEEG is also applied in preterm infants. Also, in this group aEEG predicts outcome in terms of death and disability well (97, 98). However, due to the combined outcome measure of death and disability – this implies that data on the value of aEEG and EEG to predict CP are currently lacking (99).

Additional neurophysiological methods, which are used to predict developmental outcome in high-risk infants are the visual evoked potential (VEP) and somatosensory evoked potential (SEP). SEP is recorded as a reaction to stimulation of the median nerve or the posterior tibial nerve. The review of Laerhoven et al. (95) indicated that both SEP and VEP may help to predict death or disability in infants with moderate to severe neonatal encephalopathy. However, the predictive values of SEP and VEP are less than those of aEEG and EEG. Little is known on the value of neonatal SEP and VEP to predict CP. The relatively small studies of Suppiej et al. [in infants with neonatal encephalopathy (100)] and Pike and Marlow [in preterm infants (101)] suggested that SEP presumably predicts abnormal neuromotor outcome better than VEP.

## Early Intervention in CP

The effect of early intervention has been studied predominantly in infants at high risk for developmental disorders, i.e., in groups of preterm infants only (102), or in mixed groups of high-risk infants (103). The large majority of the infants studied did, however, not develop CP. This implies that our knowledge on early intervention in infants who do develop CP is limited. I will first address the effect of early intervention in high-risk infants. Next, I will discuss the data available on early intervention in infants who later are diagnosed with CP.

### Early intervention in high-risk infants

In preterm infants intervention may start prior to term age. The limited evidence available suggests that the newborn individualized developmental care and assessment program (NIDCAP) and infant massage (103, 104) are associated with a short-term beneficial effect on brain development. However, the evidence on long-term effects of these interventions is inconclusive. On the effect of early intervention in preterm infants after term age more information is available. A meta-analysis (105) and a systematic review (102) indicated that early intervention by means of general developmental programs is associated with a positive effect on cognitive development until the age of 3 years. Whether or not the effect persists beyond this age is not clear, as few studies followed the children at school age or in adolescence. The systematic review of Spittle et al. (102) suggests that the effect of early intervention disappears after preschool age. However, follow-up of the infant behavioral assessment and intervention program (IBAIP) and the infant health and development program (IHDP) indicates that some effects of early intervention may sustain beyond preschool age (106, 107). The IBAIP program applied in very low-birth weight infants was associated with a minor advantage in performance intelligence quotient, visuomotor integration, and the ball task “aiming and catching” at the age of 5.5 years (106). The IHDP program, which was applied in low-birth weight infants, was associated with higher scores on vocabulary and mathematics tests and with less risk behavior at 18 years of age. However, these effects were found only in the subgroup of participants with a birth weight of 2000–2500 g and not in those with a lower birth weight (107). The authors suggested that adverse biological factors may offset the beneficial effect of intervention.

The early intervention studies also showed that in general the effect of developmental programs on motor development is small and does not persist beyond infancy (102, 105). Wallander et al. (108) applied the concept of early developmental intervention in asphyxiated infants in the LMIC setting. The results of this study were similar to those of the early intervention studies in preterm infants in more affluent settings: intervention promoted development until the age of 3 years, and the effect on cognitive development was larger than that on motor development. The systematic review of Blauw-Hospers and Hadders-Algra (103) paid special attention to the contents of the various early intervention programs. The review underlined two points. First, no evidence is available for a beneficial effect of early intervention by means of neurodevelopmental treatment (NDT) or Vojta therapy. Second, the general developmental programs that are associated with a positive effect on developmental outcome are very heterogeneous. This implies that our knowledge on the effective elements of intervention is limited.

Early intervention in general comprises in addition to the therapeutic developmental interventions targeting the infant, some form of parental support, including psychosocial support and parent education. As a result, general developmental programs are also associated with a reduction of maternal anxiety and depression, improved maternal self-efficacy, and – presumably – less maternal stress (109). Possibly, the effect of the programs on the mother is one of the mediators of the effect of early intervention on the infant’s development. However, the way in which parents are involved in early intervention differs considerably. Traditionally, parents have been assigned the role of co-therapist. But gradually, awareness of family autonomy arose, leaving room for individual parenting and educational styles in early intervention (110, 111). The concept of family coaching as opposed to parent training emerged (111). Family coaching in early intervention implies that families set the goals for intervention and that the coach provides – by means of an open dialog – hints and suggestions how the goals may be achieved during daily routines, such as feeding and bathing (110–112). A recent study in high-risk infants indicated that family coaching during early infancy was associated with improved motor development and functional mobility at 18 months CA (113, 114). This suggests that family coaching may be one of the potentially effective factors in family centered care.

### Early intervention in infants developing CP

Very little information is available on the effect of early intervention in the subset of high-risk infants, who later are diagnosed with CP. When I refer to this subgroup, I will use the expression “infants developing CP” or “infants who develop CP.” However, note that this label can be assigned only retrospectively. Four studies addressed the effect of general early intervention programs in infants with brain lesions on cUS (115–117) or in infants who developed CP (113, 114). Nelson et al. studied in 37 infants, the effect of auditory, tactile, visual, and vestibular stimulation provided from preterm age to 2 months CA. The intervention was not associated with a difference in motor and cognitive outcome at 12 months CA assessed with the Bayley scales of infant development (115). Follow-up data were, however, only available in 27 infants, meaning that the study suffered from the risk of selection bias due to substantial attrition and that the study was underpowered. Ohgi et al. assessed in 23 infants, the effect of a neonatal behavioral intervention starting in the preterm period and continued until 6 months CA. The behavioral intervention was associated with improved behavioral state regulation at 4 weeks CA, but no effect of intervention could be demonstrated on motor and cognitive development at 6 months CA measured with the Bayley scales of infant development (116). It should be noted, however, that the study was underpowered. Weindling et al. compared in 87 infants of whom 45 developed CP, the effect of infant physical therapy based on NDT to standard care. Motor and cognitive outcome measured with Griffiths developmental assessment of both groups was similar at 3 years of age (117). Also, this study lacked the power to demonstrate statistically significant differences. In the Netherlands, the effect of the family centered program COPCA [coping with and caring for infants with special needs (110, 111)] applied between 3 and 6 months CA was evaluated in 10 infants who developed CP. Developmental outcome at 18 months CA of the group who had received COPCA intervention did not differ from the group that had received traditional infant physical therapy. Also, this study was underpowered. In addition, quantitative video-analysis of the contents of the interventions demonstrated considerable overlap in the physiotherapeutic actions of the intervention and control group. Process evaluation based on the quantitative video information revealed that the time during intervention spent on coaching of the family and on varied motor activities during which the infants’ capacities were challenged, were associated with better motor development and functional mobility at 18 months CA (113, 114). Currently, a replication randomized controlled trial on the effect of COPCA on developmental outcome and family function in infants at very high risk for CP is carried out (118). Overall, the reviewed studies indicate that virtually no evidence is available on the effect of early intervention in infants developing CP, as the studies available were underpowered or suffered from overlap in the contents of intervention in study and control group (119, 120).

Other studies evaluated the effect of intervention in children with CP or with a high suspicion of CP, starting the intervention at about 1 year of age (range 4–36 months CA). The oldest study was performed in the seventies of last century (121). It evaluated in 24 infants whom mostly developed dyskinetic CP, whether neurophysiologically based physical therapy, including parent training and applied twice a month, affected the child’s motor development more than equally frequently applied therapy consisting of passive movements to promote joint mobility. The intervention started between 5 and 17 months (median: 11 months) and outcome was assessed at the age of 2 years. The results showed that outcome in both group was similar; however, also this study was underpowered. Mayo (122) evaluated in the eighties of last century in 29 infants who mostly developed spastic CP, whether 6 months of NDT provided once a week had a better effect on a non-validated, complex aggregated parameter of motor outcome than 6 months of NDT provided once a month. The infants entered the study between 4 and 18 months of age (median 12: months). The study indicated that the higher dosage of NDT (once a week) was associated with better motor development. The Palmer et al. study (123) was also carried out in the eighties of last century. This randomized controlled trial included 48 children aged 12–19 months with bilateral spastic CP (diplegic type). The study compared the effect of 1 year of NDT provided in two sessions per month with a home-based infant-stimulation program that included motor, sensory, language, and cognitive activities of increasing complexity. At the end of the intervention, motor development was significantly better in the children who had received the infant-stimulation program than in those who had received NDT. Cognitive development in both groups was similar. This well designed study with little attrition provided good evidence that an infant-stimulation program is associated with better motor outcome than NDT. Finally, Reddihough et al. (124) evaluated in the nineties of last century in a group of 66 children with CP, whether 6 months of conductive education provided for 8 h/week had a more beneficial effect on motor development than traditional therapy provided with a similar intensity. The children aged 12–36 months at study entry (mean: 22 months). Outcome measured with the Gross Motor Function Measure at the end of intervention was similar in both groups. Only part of the study was carried out with a randomized assignment to intervention or control group, which reduced the study’s validity. Overall, only one of the four studies, which evaluated the effect of early intervention that started after the age of 3 months CA provided evidence on the effect of early intervention; the Palmer et al. study (123) provided moderately strong evidence that intervention by means of an infant-stimulation program provided twice per month was associated with better motor outcome than equally frequent intervention by means of NDT.

Recently, a large randomized controlled trial (*n* = 128) compared the effect of 6 months of context-focused therapy with that of a similar period of child-focused therapy in preschool aged children with CP [mean age at study entry: 3.5 years (125)]. After the intervention, gross motor function and daily life function had improved in both groups, but in both groups to a similar extent. The finding of a similar outcome in this high-quality study is intriguing, as the theoretical background of both approaches is substantially different – in the context-focused therapy the family and functional performance are key notions in the intervention, whereas the child-focused approach emphases improvement of the child’s movement skills. The outcome of the study stresses the need of detailed process evaluation in early intervention studies, and the need of interventions that are tailored to the specific impairments of the child and the specific needs and wishes of the family. Goal directed functional therapy may offer one of the means to improve the success of early intervention (126, 127).

Next to the more general programs of early intervention, also specific interventions have been developed. For instance, Mattern-Baxter et al. (128) recently studied the effect of 6 weeks of intensive home-based treadmill training in 12 young children with CP (aged 9–36 months, mean: 21 months; Gross Motor Function Classification System levels I and II). The intervention group of six infants received twice daily during 6 days/week treadmill training sessions of 10–20 min. During the intervention period of 6 weeks, the infants continued to receive their usual therapy. The six control infants received only their usual therapy. Direct after the intervention, but also 4 months post-intervention function in daily life of the infants who had received treadmill training was significantly better than that of the control group. Currently, other specific approaches of early intervention are studied. The upper limb baby early action-observation training (UP-BEAT) study is an example (129). It studies the effect of action–observation training in infants with an asymmetric brain lesion, as action–observation has been shown to promote bimanual function in older children with unilateral CP (130). Action observation is based on the idea that new motor skills can be learned by observing motor actions, a coupling, which is facilitated by the function of the mirror neuron system (131). Another promising approach is the application of constraint-induced movement therapy (CIMT) in infancy [Baby-CIMT (132)]. CIMT is known to be effective in promoting bimanual activities in older children with unilateral CP, an effect, which, however, also may be achieved by bimanual training (133).

## Concluding Remarks

The lesions of the brain underlying CP vary substantially in size, site, and time of occurrence. Most lesions occur between 24 weeks PMA and term age, a period during which developmental activity in the brain reaches its summit. Variations in timing of the insult to the brain not only result in different lesions but also in different neuroplastic reactions and different associated neuropathologies. This turns CP into a very heterogeneous entity. This may mean that the best early diagnostics and the best intervention methods may differ for various subgroups of children with CP.

Currently, prediction of CP in early infancy is best when based on multiple assessment techniques and series of assessments. In infants admitted to a NICU, the combination of neonatal imaging of the brain and GMA results in best prediction of CP. In infants who are not admitted to neonatal intensive care services, careful documentation of milestones in combination with a neurological assessment currently is the best – but non-optimal way – to detect infants developing CP.

Knowledge on the effect of early intervention in infants with severe CP is conspicuous by its absence. Animal studies suggest that the opportunities for a beneficial effect of intervention after a severe lesion of the brain are limited (33, 34). This does not mean that hope for improvement of function is nil. But, it may mean that we should encourage families to use already at early age assistive devices. Examples are adaptive seating devices, which promote upright sitting and therewith a better orientation in the environment (134), and power mobility, such as modified ride-on toy cars, allowing for exploration of the environment (135). The assistive devices may promote the children’s social and cognitive development (136). Information on the effect of the assistive devices is urgently needed.

The studies in high-risk infants without CP indicate that early intervention has a stronger effect on cognitive development than on motor outcome. This result is in line with the animal studies on early intervention (33, 34). The effect on cognitive outcome is, however, essential as cognitive function determines to a much larger extent activities and participation of children with CP than motor function (137, 138).

The early occurrence of the lesion of the brain in CP offers opportunities for early intervention. The evidence that early intervention is able to improve developmental outcome in children with CP is, however, very limited. Weak evidence suggests that parental coaching and provision of hints and suggestions on how to challenge infant activities during daily life – characteristic elements of the COPCA program (110, 111) – are associated with improved functional outcome. Moderate evidence indicates that in infants older than 1 year infant stimulation has a better effect on motor development than NDT. On the other hand, weak evidence suggests that a higher dosage of NDT used in infants older than 1 year may result in a better outcome than a lower dosage. In older children with CP, it is well-known that high dosages of therapy have a better effect than low dosages (133). The most elegant and most efficient way to achieve high dosages of specific activities is by integrating practice into daily life activities. This underscores the need of family centered early intervention. This may not only improve body function but, more importantly, also activities and participation.

## Conflict of Interest Statement

The author declares that the research was conducted in the absence of any commercial or financial relationships that could be construed as a potential conflict of interest.
